# Systemic Inflammatory Markers in Parkinson’s Disease: Correlations with Disease Severity and Neuropsychiatric Symptoms

**DOI:** 10.3390/brainsci16101133

**Published:** 2026-10-20

**Authors:** Meimei Zhang, Wei Li, Bei Kang, Yumei Zhang, Tao Feng

**Affiliations:** 1 Department of Neurology, Shaanxi Provincial People’s Hospital, Xi’an 710068, China; 2 China National Clinical Research Center for Neurological Diseases (NCRC-ND), Beijing 100070, China; 3 Department of Rehabilitation, Beijing Tiantan Hospital, Capital Medical University, Beijing 100070, China; 4 Department of Neurology, Beijing Tiantan Hospital, Capital Medical University, Beijing 100070, China

**Keywords:** Parkinson’s disease, neuroinflammation, systemic inflammation, inflammatory indices

## Abstract

**Highlights:**

**What are the main findings?**

- SIRI, MLR, and MHR were elevated in PD versus HC, but SII and PLR were not independently associated with disease severity after comprehensive adjustment.
- Disease duration and hypertension, rather than inflammatory indices, were the strongest correlates of higher H–Y staging.

**What are the implications of the main findings?**

- Peripheral inflammatory indices are altered in PD but not independently linked to disease severity or symptoms after comprehensive adjustment.
- Previously reported severity associations may reflect confounding by disease duration and vascular comorbidity.

**Abstract:**

**Objective:** Chronic neuroinflammation is a recognized driver of Parkinson’s disease (PD) pathophysiology, in which systemic inflammation may also participate. This study evaluated composite inflammatory indices in peripheral blood and investigated their associations with disease severity, motor symptoms, and non-motor symptoms. **Methods:** A retrospective study was conducted using routinely collected clinical and laboratory data. A total of 315 PD patients and 125 healthy controls (HCs) were enrolled. All PD patients underwent motor and non-motor symptom assessments. Differences in peripheral inflammatory indices were compared between the PD and HC groups, and across Hoehn–Yahr (H–Y) stages. Multinomial logistic regression was used for factors associated with disease severity. Multivariable linear regression examined correlations with clinical measures. Benjamini–Hochberg false discovery rate (FDR) correction was applied to PD vs. HC comparisons, H–Y group comparisons, and all symptom-related analyses. **Results:** Levels of the systemic inflammation response index (SIRI), monocyte-to-lymphocyte ratio (MLR), and monocyte-to-high-density lipoprotein cholesterol ratio (MHR) were significantly elevated in PD after FDR correction. In the multinomial logistic regression model, disease duration was significantly associated with both H–Y < 3 vs. H–Y > 3 (OR = 0.940, 95% CI: 0.894, 0.989; *p* = 0.016) and H–Y = 3 vs. H–Y > 3 (OR = 0.961, 95% CI: 0.925, 0.999; *p* = 0.043). Hypertension was associated only with H–Y < 3 vs. H–Y > 3 (OR = 0.468, 95% CI: 0.226, 0.970; *p* = 0.041). Platelet-to-lymphocyte ratio (PLR) was significantly associated with H–Y = 3 vs. H–Y > 3 (OR = 0.989, 95% CI: 0.980, 0.997; *p* = 0.012) but not with H–Y < 3 vs. H–Y > 3 (*p* = 0.167). Systemic immune-inflammation index (SII) was not significantly associated with either comparison. SII × sex (*p* = 0.496) and PLR × sex (*p* = 0.048, Bonferroni-corrected *p* > 0.025) interaction terms were not significant. After FDR correction, no inflammatory indices were significantly associated with motor or non-motor symptom (all q > 0.05). **Conclusions:** After rigorous adjustment, previously reported associations between peripheral inflammatory indices and PD severity were not replicated. Disease duration and hypertension were the primary correlates of higher H–Y staging. Elevated inflammatory indices should be interpreted with caution, as they may be associated with longer disease duration or vascular comorbidity.

## 1. Introduction

Parkinson’s disease (PD), the second most common neurodegenerative disorder, involves the deposition of misfolded α-synuclein (α-syn) and loss of dopaminergic neurons in the substantia nigra. PD is projected to pose a growing public health challenge for patients, caregivers, and society by 2050 [1]. Its clinical presentation includes motor symptoms—such as resting tremor, bradykinesia, rigidity, and gait/postural instability—together with non-motor symptoms like cognitive impairment, emotional disorders, and autonomic dysfunction.

Despite decades of research, the precise etiology and pathogenic mechanisms of PD remain incompletely understood. Neuroinflammation is widely recognized as a key driver of PD pathophysiology [2]. Within the central nervous system (CNS), the activation of microglial cells is the primary inflammatory response [3]. Aggregated α-syn stimulates excessive microglial activation, thereby promoting a more active inflammatory response [4,5,6] and subsequent neuronal damage. Evidence indicates that the peripheral immune system communicates with the CNS via a disrupted blood–brain barrier, glymphatic system, and meningeal lymphatic vessels [7,8,9], suggesting that systemic inflammation may contribute to neuroinflammation in PD.

In recent years, the convenient detection and low cost of peripheral blood inflammatory markers have made them a focus of research. Unlike single indicators, composite inflammatory indices more comprehensively reflect immune and inflammatory status. Among these, neutrophil- and lymphocyte- based ratios, including systemic immune-inflammation index (SII), systemic inflammation response index (SIRI), neutrophil-to-lymphocyte ratio (NLR), platelet-to-lymphocyte ratio (PLR), monocyte-to-lymphocyte ratio (MLR), and neutrophil-to- platelet ratio (NPR), capture different facets of the innate-adaptive immune balance and have been widely studied in inflammatory disease [10]. Additionally, high-density lipoprotein cholesterol (HDL-C)-based ratios, including neutrophil-to-high-density lipoprotein cholesterol ratio (NHR), monocyte-to-high-density lipoprotein cholesterol ratio (MHR), lymphocyte-to-high-density lipoprotein cholesterol ratio (LHR), and platelet-to-high-density lipoprotein cholesterol ratio (PHR), have drawn attention because HDL-C possesses antioxidant and anti-inflammatory properties and exhibits an inverse association with systemic inflammation and endothelial dysfunction [11]. These may provide a more integrated measure of lipid-associated inflammation. Studies have shown that NHR and MHR may serve as indicators of peripheral inflammation in PD [12,13]. Thus, the ten indices were selected to systematically cover both cell-based and lipid-integrated peripheral inflammatory metrics, allowing a comprehensive evaluation of different inflammatory dimensions in PD.

However, current findings regarding changes in these composite inflammatory indices in patients with PD remain inconsistent. Most studies have been limited to investigating their association with the risk of PD [14,15], while systematic clinical data are still lacking on the correlation between these indices and disease severity, motor or non-motor symptoms. Moreover, prior studies have been limited by inadequate adjustment for key confounders such as disease duration, use of levodopa, and vascular comorbidities (hypertension, diabetes), all of which influence both inflammatory indices and clinical outcomes [16,17]. Additionally, few studies have formally validated biomarker–sex interactions, and the distinction between disease-specific versus physiological differences remains unclear.

Therefore, the purpose of this study is to assess the ten peripheral blood composite inflammatory indices above-mentioned in PD patients and to investigate their associations with disease severity, motor symptoms, and non-motor symptoms, with comprehensive adjustment for potential confounders. We also examined sex differences in these inflammatory indices and formally tested biomarker–sex interactions. Based on prior literature, SII and PLR were selected as primary exposure markers for severity of PD [18,19]. Because this selection was literature-driven rather than prespecified, these analyses were considered exploratory, as were the remaining eight indices and all sex-stratified and symptom-related analyses.

## 2. Materials and Methods

### 2.1. Study Design and Participants

A retrospective study was conducted using routinely collected clinical and laboratory data from patients hospitalized at the Department of Neurology of Beijing Tiantan Hospital, Capital Medical University, between January 2019 and September 2022 (PD group). This study is reported in accordance with the Reporting of studies Conducted using Observational Routinely-collected Data (RECORD) guidelines (http://www.record-statement.org, accessed on 16 October 2026) [20]. Sample size was not pre-calculated due to retrospective consecutive enrollment. Patients enrolled met the following inclusion criteria: (1) clinically definite PD according to the clinical diagnosis criteria published in 2015 [21]; (2) completion of relevant clinical and laboratory examinations. Exclusion criteria included: (1) a diagnosis of Parkinsonism or Parkinson-plus syndromes; (2) presence of other neurodegenerative conditions, such as Alzheimer’s disease or frontotemporal dementia; (3) comorbidities such as hypothyroidism, tumor, acute infection, severe liver or kidney injuries, and blood immune system diseases; (4) a history of stroke or head trauma.

Healthy controls (HCs) were recruited from the Health Examination Center of Beijing Tiantan Hospital between January 2020 and January 2022. Inclusion criteria were: no neurological or psychiatric disorders; no active inflammatory, infectious, or autoimmune diseases; and no use of anti-inflammatory or immunosuppressive medications within 1 month before enrollment. HCs were not individually matched to PD patients; however, the two groups had comparable age and sex distributions (*p* > 0.05). This study was carried out in compliance with the Declaration of Helsinki and authorized by Beijing Tiantan Hospital’s Ethics Committee.

### 2.2. Clinical Assessment

At admission, demographic information, including age, sex, body mass index (BMI), years of education, age at onset, and disease duration, was collected. Medical history, including hypertension, diabetes, smoking, drinking, and antiplatelet agents, was also collected. H–Y staging was used to objectively assess the disease severity of PD. Motor symptoms were quantitatively evaluated by standardized trained clinical raters who were blinded to laboratory results, utilizing Part III of the Movement Disorder Society Unified Parkinson’s Disease Rating Scale (MDS-UPDRS III). For non-motor symptoms, the Montreal Cognitive Assessment (MoCA) was used to evaluate overall cognitive function, and the Hamilton Anxiety Scale (HAMA) and Hamilton Depression Scale (HAMD) were applied to assess emotional status. The evaluations were conducted in the “ON state” (1 to 2 h after medication intake). The daily dose equivalent of levodopa (LEDD) was calculated using standard protocols [22].

### 2.3. Laboratory Data Collection

After 8 h of fasting, blood samples were collected in the early morning. Total leukocyte count, lymphocyte count, neutrophil count, monocyte count, platelet count, and HDL-C were measured using a Sysmex XN-20 automated hematology analyzer (Sysmex Corporation, Kobe, Japan) (for blood counts) and Roche Cobas 8000 (Roche Diagnostics GmbH, Mannheim, Germany) (for HDL-C). Units were as follows: leukocytes, lymphocytes, neutrophils, monocytes, and platelets ×10^9^/L; HDL-C mmol/L. Quality control included daily internal quality control and regular external quality assessment.

Based on the original single indicators above, we calculated the following composite inflammatory indices: NLR, PLR, MLR, NPR, NHR, LHR, MHR, PHR, SII (calculated as neutrophils × platelets/lymphocytes), and SIRI (calculated as (neutrophils × monocytes/lymphocytes). These composite inflammatory indices were included in the final analysis.

### 2.4. Variables Definition

Exposure variables: composite inflammatory indices (SII, SIRI, NLR, PLR, MLR, NPR, NHR, LHR, MHR, PHR).

Outcome variables: H–Y staging, MDS-UPDRS III, MoCA, HAMA, HAMD.

Confounders: age, sex, disease duration, BMI, LEDD, hypertension, diabetes, smoking, drinking, antiplatelet agents, and years of education (for MoCA).

### 2.5. Data Access, Cleaning, and Linkage

The investigators had full access to de-identified person-level clinical and laboratory data used in this study. Standard data cleaning was performed before analysis. Records were excluded if they were invalid, duplicate, or incomplete. Incomplete records were defined as missing key demographic, clinical, or laboratory variables required for the analyses. All analyses were based on complete cases. No database linkage was performed in this study.

### 2.6. Statistical Analyses

All statistical procedures were conducted using SPSS 26.0. Normality of the data was checked via the Shapiro–Wilk test. Depending on the distribution, continuous variables were described as mean and standard deviation (SD) or median and interquartile range (IQR), while categorical variables were reported as counts (percentages). Group comparisons between HC and PD were conducted using the Student’s *t*-test or Mann–Whitney U test. For laboratory indices across different H–Y stages in PD, one-way ANOVA or the Kruskal–Wallis H test was applied. Benjamini–Hochberg false discovery rate (FDR) correction was applied separately to the PD vs. HC comparisons across the ten inflammatory indices and to the H–Y group comparisons across the ten indices; q < 0.05 was considered significant.

Because the proportional odds assumption was violated (test of parallel lines, *p* < 0.001), multinomial logistic regression was used as the primary analysis to examine factors associated with H–Y staging, with H–Y > 3 as the reference category. Odds ratios (OR) with 95% confidence intervals (CI) were reported. Covariates included age, sex, disease duration, BMI, LEDD, hypertension, diabetes, smoking, drinking, and antiplatelet agents.

To assess whether sex modified the associations, two sets of interaction tests were performed. First, for inflammatory indices that showed significant sex differences in the sex-stratified comparisons, PD/HC group × sex interactions were further tested using linear regression, with Bonferroni correction for the number of indices tested. Second, SII × sex and PLR × sex interactions were tested in separate ordinal regression models for the severity analysis.

Associations between inflammatory indices and motor or non-motor symptoms were evaluated by multivariable linear regression analysis. Unstandardized regression coefficient (B) and 95% CI were reported. All models were adjusted for age, sex, disease duration, and LEDD; MoCA models were additionally adjusted for years of education. Benjamini–Hochberg FDR correction was applied for the multiple comparisons (q < 0.05 was considered significant). All statistical tests were two-tailed, and *p* < 0.05 was considered statistically significant.

## 3. Results

### 3.1. Characteristic of Participants

**Figure 1:**
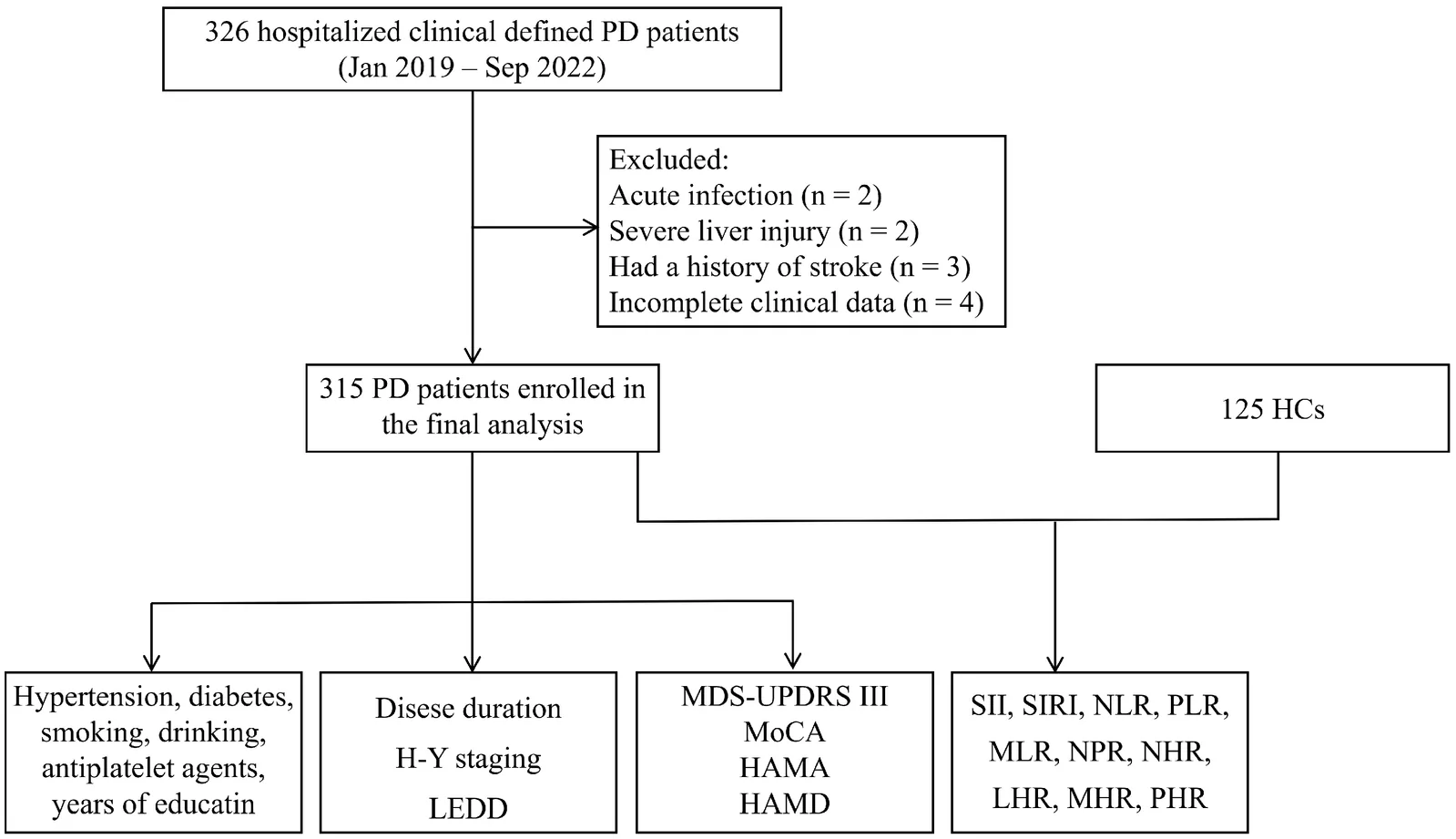
Flow diagram of participant screening and enrollment. Abbreviations: PD, Parkinson’s disease; HC, healthy control; H–Y, Hoehn–Yahr; LEDD, levodopa equivalent daily dosage; MDS-UPDRS III, Movement Disorder Society-Unified Parkinson’s Disease Rating Scale part III; MoCA, Montreal Cognitive Assessment; HAMA, Hamilton Anxiety Scale; HAMD, Hamilton Depression Scale; SII, systemic immune-inflammation index; SIRI, systemic inflammation response index; NLR, neutrophil-to-lymphocyte ratio; PLR, platelet-to-lymphocyte ratio; MLR, monocyte-to-lymphocyte ratio; NPR, neutrophil-to-platelet ratio; NHR, neutrophil-to-high-density lipoprotein cholesterol ratio; MHR, monocyte-to-high-density lipoprotein cholesterol ratio; LHR, lymphocyte-to-high-density lipoprotein cholesterol ratio; PHR, platelet-to-high-density lipoprotein cholesterol ratio.

After excluding 2 with acute infection, 2 with severe liver injury, 3 with a history of stroke, and 4 who lacked clinical data, a total of 315 PD patients (184 males and 131 females) with a mean age of 63.16 ± 8.94 years were enrolled (Figure 1). The HC group comprised 125 individuals (63 males and 62 females) with a mean age of 64.27 ± 6.98 years. No significant differences in age or sex were found between the two groups (*p* > 0.05; Table 1). PD patients had a median disease duration of 6.00 years and a median H–Y staging of 3.00.

### 3.2. Comparison of Laboratory Indices Between PD and HC

Significant differences in SIRI (Z = −2.805, *p* = 0.005), MLR (Z = −5.596, *p* < 0.001), and MHR (Z = −5.828, *p* < 0.001) were observed between the PD and HC groups (Table 1). After Benjamini–Hochberg FDR correction across the ten indices, SIRI (q = 0.017), MLR (q < 0.001), and MHR (q < 0.001) remained significantly different between PD and HC; none of the other indices survived correction (Supplementary Table S1).

**Figure 2:**
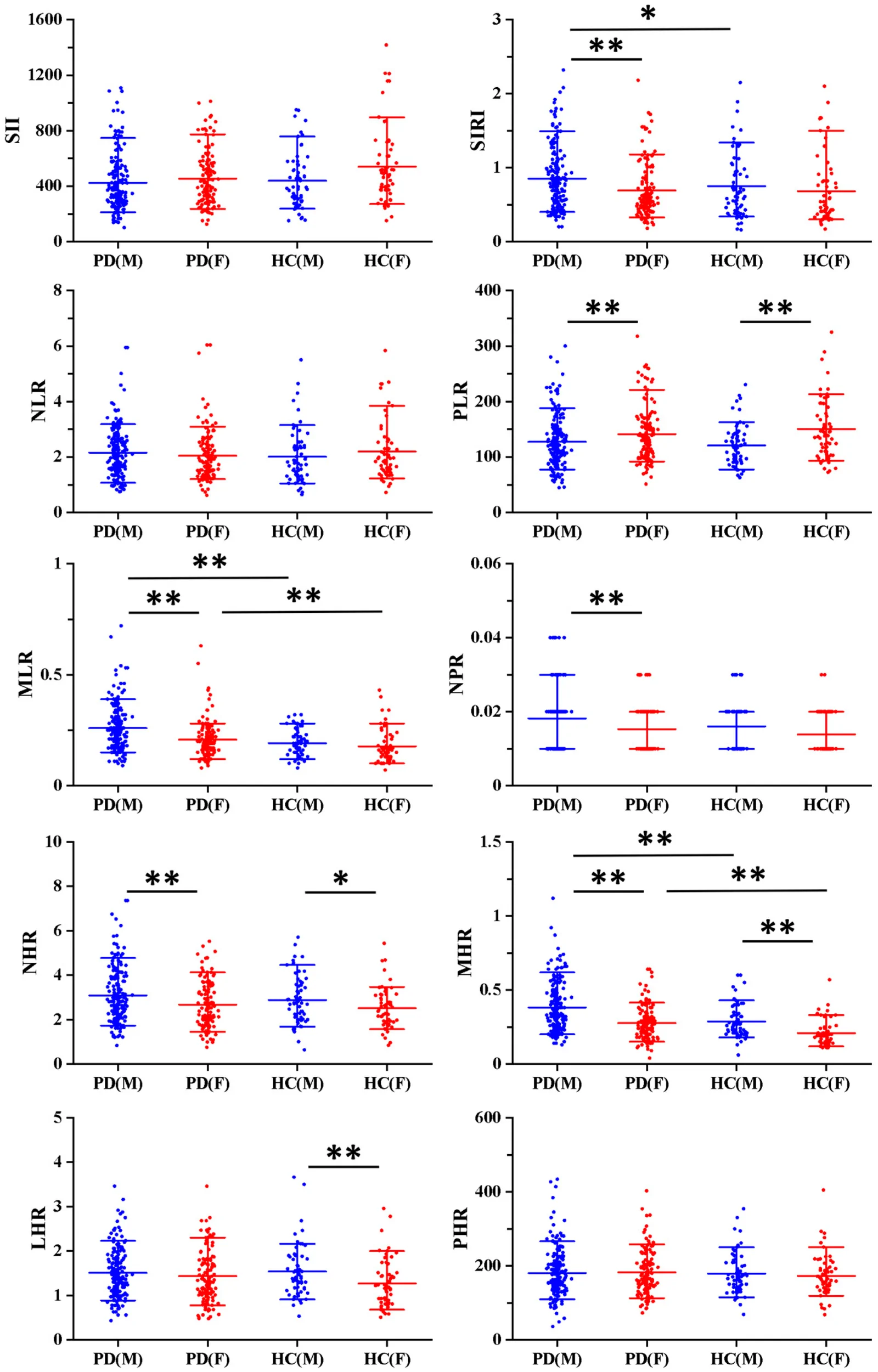
Comparison of differences in laboratory indices between PD and HC by sex. * *p* < 0.05, ** *p* < 0.01. Sex-stratified comparisons were exploratory and not corrected for multiple testing. Abbreviations: PD(M), males with Parkinson’s disease; PD(F), females with Parkinson’s disease; HC(M), healthy control males; HC(F), healthy control females; SII, systemic immune-inflammation index; SIRI, systemic inflammation response index; NLR, neutrophil-to-lymphocyte ratio; PLR, platelet-to-lymphocyte ratio; MLR, monocyte-to-lymphocyte ratio; NPR, neutrophil-to-platelet ratio; NHR, neutrophil-to-high-density lipoprotein cholesterol ratio; MHR, monocyte-to-high-density lipoprotein cholesterol ratio; LHR, lymphocyte-to-high-density lipoprotein cholesterol ratio; PHR, platelet-to-high-density lipoprotein cholesterol ratio.

Given the potential influence of sex, we performed a subgroup comparison. Figure 2 presents sex-specific differences in laboratory indices between PD patients and HCs. Among the male participants, SIRI, MLR, and MHR were significantly different between PD and HC (*p* < 0.05, *p* < 0.01). In female participants, only MLR and MHR showed significant differences (*p* < 0.01). Male PD patients exhibited higher levels of SIRI, MLR, NPR, NHR, and MHR than female PD patients, whereas PLR was higher in female patients (*p* < 0.01, Figure 2). Among the HCs, males exhibited higher levels of NHR, MHR, and LHR than females, while PLR was again higher in females (*p* < 0.01, Figure 2).

We further formally tested the group × sex interaction for the indices that showed significant differences in Figure 2 (SIRI, PLR, MLR, NPR, NHR, and MHR). Given that six interaction tests were performed, we applied Bonferroni correction (threshold *p* < 0.008). None of the interaction terms were significant after correction for multiple comparisons (all Bonferroni-corrected *p* > 0.008; Supplementary Table S2), indicating no statistical evidence for sex-specific effects. The sex-stratified comparisons in Figure 2 should therefore be regarded as exploratory and descriptive.

### 3.3. Comparison of Laboratory Indices Among H–Y Staging in PD

We stratified PD patients into three groups based on the H–Y staging: H–Y < 3 (*n* = 102), H–Y = 3 (*n* = 132), and H–Y > 3 (*n* = 81). ANOVA or Kruskal–Wallis H test revealed significant differences in SII and PLR among these groups (*p* < 0.05, Table 2). However, after Benjamini–Hochberg FDR correction across the ten indices, neither difference remained statistically significant (all q > 0.05, Supplementary Table S1).

### 3.4. Correlation Between Laboratory Indices and Disease Severity of PD

Because the proportional odds assumption was violated (*p* < 0.001), multinomial logistic regression was used as the primary analysis with H–Y > 3 as the reference category (Table 3). Adjusted for age, sex, BMI, LEDD, hypertension, diabetes, smoking, drinking, and antiplatelet agents, disease duration was associated with lower odds of both H–Y < 3 (OR = 0.940, 95% CI: 0.894, 0.989; *p* = 0.016) and H–Y = 3 (OR = 0.961, 95% CI: 0.925, 0.999; *p* = 0.043) relative to H–Y > 3. Hypertension was associated only with H–Y < 3 vs. H–Y > 3 (OR = 0.468, 95% CI: 0.226, 0.970; *p* = 0.041); BMI (OR = 0.917, 95% CI: 0.846, 0.994; *p* = 0.035) and antiplatelet use (OR = 1.311, 95% CI: 0.842, 2.259; *p* = 0.012) were associated only with H–Y = 3 vs. H–Y > 3. PLR was associated with H–Y = 3 vs. H–Y > 3 (OR = 0.989, 95% CI: 0.980, 0.997; *p* = 0.012) but not H–Y < 3 vs. H–Y > 3 (*p* = 0.167). SII was not associated with either comparison (both *p* > 0.05), and LEDD showed a trend for H–Y < 3 vs. H–Y > 3 (*p* = 0.050).

SII × sex (*p* = 0.496) and PLR × sex (*p* = 0.048, Bonferroni-corrected *p* > 0.025) interactions were not significant.

In multinomial logistic regression models for the remaining eight indices (Supplementary Table S3), NLR was significantly associated with both H–Y < 3 vs. H–Y > 3 (OR = 0.736, 95% CI: 0.541, 1.000; *p* = 0.050) and H–Y = 3 vs. H–Y > 3 (OR = 0.753, 95% CI: 0.576, 0.985; *p* = 0.039). The other seven indices were not significantly associated with H–Y staging (all *p* > 0.05).

### 3.5. Correlation Between Laboratory Indices and Clinical Symptoms in PD

In univariate and minimally adjusted (age + sex) linear regression analyses, SII, SIRI, PLR, MLR, and PHR each showed nominally significant associations with at least one clinical outcome (Supplementary Table S4). In fully adjusted models (Table 4), SII was associated with MoCA scores (B = −0.003, 95% CI: −0.005, 0.000; *p* < 0.05) and HAMD (B = 0.006, 95% CI: 0.001, 0.010; *p* < 0.05). SIRI was associated with MoCA (B = −0.948, 95% CI: −1.881, −0.014; *p* < 0.05). PLR was associated with MDS-UPDRS III (B = 0.049, 95% CI 0.008, 0.091; *p* < 0.05), HAMA (B = 0.024, 95% CI 0.003, 0.045; *p* < 0.05), and HAMD (B = 0.034, 95% CI 0.009, 0.059; *p* < 0.01). After Benjamini–Hochberg FDR correction across all ten indices and four outcomes (40 tests), none of these associations remained statistically significant (all q > 0.05; Supplementary Table S5). These findings should be considered exploratory.

## 4. Discussion

The current study systematically investigated the association between novel composite inflammatory indices and disease severity, motor and non-motor symptoms in PD patients by analyzing peripheral blood inflammatory indices. Our principal findings were as follows: (1) after FDR correction, SIRI, MLR, and MHR remained significantly elevated in the PD group compared to the HC group; (2) disease duration and hypertension were the strongest correlates of higher H–Y staging; (3) after FDR correction, no inflammatory index was significantly associated with motor or non-motor symptoms of PD; and (4) formal interaction tests did not support sex-specific effects. Collectively, these findings indicate that peripheral inflammatory indices are altered in PD but are not independently associated with disease severity or clinical symptoms, highlighting the importance of accounting for clinical confounders.

Our study observed that levels of SIRI, MLR, and MHR were significantly elevated in PD patients after FDR correction, consistent with previous reports [13,23,24]. Some investigations have noted only a non-significant upward trend in these markers [12,25]. Although elevated NLR, NHR, and PLR have been described in PD patients [18,26], we observed no significant intergroup differences for these indices. Racial differences within the studied cohorts may explain these divergent results [27].

Notably, the subgroup comparison by sex revealed distinct patterns. Compared to HC, SIRI, MLR, and MHR were significantly elevated in males with PD, whereas only MLR and MHR differed significantly in females. Males exhibited higher levels of NHR and MHR than females in both PD and HC, while PLR was higher in females of both groups. SIRI, MLR, and NPR showed sex differences only in PD patients. Bovenzi and his colleagues reported that NLR and MLR were significantly higher in male PD patients than in male controls and female PD patients, with cluster analysis showing that female PD patients primarily aligned with a “low peripheral inflammation” cluster, while males predominantly belonged to a “high peripheral inflammation” cluster [28]. Some of these sex differences were also partially observed in HC, suggesting that some patterns may reflect baseline physiological variations rather than PD-specific pathology. Formal interaction tests for the six indices with significant differences in Figure 2 were not significant after Bonferroni correction. These findings should therefore be considered exploratory and descriptive.

Contrary to some previous reports, SII was not independently associated with H–Y staging after comprehensive adjustment for confounders in the multinominal model. PLR was significantly associated only with the H–Y = 3 vs. H–Y > 3 comparison; however, the effect size was very small and should be interpreted with caution. One recent study identified a correlation between SII and H–Y staging using partial correlation analyses adjusted for age [19], while another linked SII to motor progression [29]. Stanca et al. reported a significant association between PLR and H–Y staging after adjustment for age [18]. This discrepancy likely reflects more stringent covariate adjustment in our analysis. Many prior studies did not adequately account for disease duration, hypertension, diabetes, or LEDD, yet these factors are intertwined with both inflammatory indices and clinical progression [16,17].

In our fully adjusted model, disease duration and hypertension were the strongest independent correlates of higher H–Y staging. The dominant role of disease duration aligns with the well-established progressive nature of PD. The association between hypertension and higher H–Y staging is consistent with previous findings linking vascular comorbidities to more severe PD [30,31]. Hypertension has been proposed to influence neurodegeneration through endothelial dysfunction, blood–brain barrier disruption, and oxidative stress [32,33], although our cross-sectional design cannot establish causality. Additionally, hypertension itself drives platelet activation and alters leukocyte counts [34,35], raising the possibility that previously reported associations between platelet-related indices (SII, PLR) and PD severity may have been confounded by vascular comorbidity rather than reflecting PD-specific neuroinflammation. This also helps explain why SII and PLR lost significance after adjustment for hypertension and other confounders in our study.

Given the observed sex differences in inflammatory indices, we further examined whether sex modified the association between SII or PLR and H–Y staging. However, the SII × sex and PLR × sex interaction terms were not significant, indicating no statistical evidence for sex-specific effects in the severity models.

In the exploratory multinomial analyses of the remaining eight indices, NLR was significantly associated with higher H–Y staging, whereas the other seven indices were not. However, these analyses were exploratory and not corrected for multiple comparisons; therefore, the NLR finding should be interpreted with caution and requires confirmation in independent cohorts.

Overall, the elevated levels of SII and PLR observed in PD with higher H–Y staging should be interpreted with caution, as they may reflect longer disease duration, vascular comorbidity, or medication effects rather than PD-specific inflammatory activity. Given the retrospective design, these findings are descriptive and exploratory, and do not establish clinical utility.

In univariate and minimally adjusted analyses, SII, SIRI, and PLR showed nominal associations with motor, cognitive, and mood scores among PD patients. However, after FDR correction for multiple comparisons, none of these associations remained statistically significant. The absence of robust associations after correction may reflect true absence of effect, insufficient statistical power, or residual confounding from unmeasured factors such as sleep quality, socioeconomic status, or medication-related mood effects, which were not available in our retrospective dataset.

The literature remains conflicting regarding the correlation between peripheral inflammatory indices and clinical symptoms in PD. One study demonstrated that SII correlated with the UPDRS total scores and its subscales [19]. Another found a positive linear association of both SII and SIRI with anxiety levels [36], while Yang’s study showed increased NLR as an indicator of worsening anxiety [37]. Two longitudinal studies reported that NLR could predict cognitive decline in PD [38,39]. In contrast, Alagoz’s study found no significant relationship between inflammatory markers and motor or non-motor symptoms [25]. Our results, which are consistent with the latter group of studies after correction, highlight that differences in study design, covariate adjustment, and correction for multiple comparisons may contribute to the heterogeneity of findings across studies.

Our study employed several methodological approaches that strengthen the robustness of our findings. First, we used multinomial logistic regression as the primary analysis for H–Y staging, avoiding the equal-interval assumption of linear regression and providing category-specific estimates. Second, we comprehensively adjusted for key confounders that were often omitted in prior studies. Third, we applied an FDR correction across all biomarker-outcome comparisons to control for multiple comparisons and reduce the risk of false-positive findings. These methodological differences likely account for the discrepancies between our results and earlier reports.

Our study complements the existing literature by systematically evaluating these indices with comprehensive adjustment for disease duration, LEDD, hypertension, diabetes, smoking and drinking, which were often inadequately controlled in prior studies. Our findings suggest that previously reported associations between SII/PLR and PD severity may have been confounded by these factors, offering an important methodological caution for future studies.

Several limitations of this study warrant consideration. First, the retrospective, single-center design and the exclusive enrollment of hospitalized patients limit the generalizability of our findings to community-dwelling or early-stage PD populations, potentially introducing selection and referral biases. Additionally, the cross-sectional nature of this study precludes any causal inferences. Although we excluded participants with incomplete records, we cannot fully rule out missing-data bias, as the reasons for missingness were not systematically recorded and may be related to patient characteristics or disease severity. Second, PD diagnosis was based on clinical criteria, which carries a risk of misdiagnosis, particularly in early stages [40]. This diagnostic uncertainty may introduce heterogeneity into the PD group, potentially diluting true associations or biasing effect estimates. Furthermore, the absence of a disease control group like atypical parkinsonism precludes determination of whether our findings are specific to PD. Future studies incorporating α-synuclein-based biomarkers may yield greater diagnostic precision [41,42]. Third, residual confounding cannot be excluded. Although we adjusted for key confounders in the PD cohort analyses, the PD vs. HC comparisons were not adjusted for vascular or metabolic factors because these data were not available for the HC group. In addition, data on several other potential confounders (e.g., statin use, cardiovascular disease history, NSAID use, dietary habits, and physical activity) and on subclinical inflammatory conditions were lacking. These unmeasured factors may have influenced our estimates. Fourth, single-time-point blood sampling may not capture stable inflammatory signatures due to circadian rhythms, transient stress, or hydration status. Longitudinal sampling is needed to determine whether these markers consistently track disease progression. Fifth, we relied on hematological ratios rather than direct cytokine measurements (IL-2, IL-6, TNF-α) [43] or immune phenotyping. Consequently, the study cannot offer a molecular-level mechanistic explanation. Addressing these limitations in future prospective, multicenter, and longitudinal studies will be important for clarifying the role of peripheral inflammation in PD.

## 5. Conclusions

In summary, after rigorous adjustment and FDR correction, disease duration and hypertension were the strongest correlates of PD severity, while SII and PLR were not independently associated. These findings suggest that previously reported associations may be confounded by disease chronicity and vascular comorbidity. Clinicians should interpret elevated inflammatory indices with caution, as they may be associated with longer disease duration or vascular risk factors, and should not be assumed to reflect PD-specific neuroinflammation.

## Figures and Tables

**Table 1:** Comparison of laboratory indicators between PD patients and HC.

	PD (*n* = 315)	HC (*n* = 125)	t/Z	*p*
Age, years	63.16 ± 8.94	64.27 ± 6.98	1.245	0.214
Sex, male/female, *n*	184/131	63/62	N/A	0.127
BMI, kg/m^2^	24.22 (21.99, 26.47)	24.51 (22.34, 26.82)	0.872	0.383
Hypertension, *n* (%)	96 (30.48%)		N/A	N/A
Diabetes, *n* (%)	38 (12.06%)		N/A	N/A
Smoking, *n* (%)	93 (29.52%)		N/A	N/A
Drinking, *n* (%)	76 (24.13%)		N/A	N/A
Antiplatelet agents, *n* (%)	25 (7.94%)		N/A	N/A
Age at onset, years	57.00 (50.00, 63.00)		N/A	N/A
Disease duration, years	6.00 (4.00, 10.00)		N/A	N/A
LEDD	500.00 (300.00, 700.000)		N/A	N/A
H–Y staging	3.00 (2.00, 4.00)		N/A	N/A
MDS-UPDRS III	39.00 (28.00, 52.00)		N/A	N/A
SII	451.11 (307.69, 578.63)	394.35 (287.93, 549.10)	−1.695	0.090
SIRI	0.69 (0.48, 1.02)	0.58 (0.38, 0.93)	−2.805	**0.005**
NLR	1.99 (1.53, 2.55)	1.84 (1.38, 2.52)	−1.027	0.304
PLR	125.00 (99.66, 154.05)	125.38 (99.30, 156.80)	−0.486	0.627
MLR	0.22 (0.17, 0.28)	0.17 (0.13, 0.22)	−5.596	**<0.001**
NPR	0.02 (0.01, 0.02)	0.01 (0.01, 0.02)	−1.712	0.087
NHR	2.71 (2.05, 3.69)	2.66 (1.94, 3.27)	−1.661	0.097
MHR	0.31 (0.23, 0.41)	0.22 (0.18, 0.32)	−5.828	**<0.001**
LHR	1.39 (1.06, 1.81)	1.32 (0.96, 1.76)	−1.353	0.176
PHR	172.95 (143.02, 209.48)	165.87 (135.69, 208.64)	−0.938	0.348

Statistically significant results are shown in bold. Abbreviations: PD, Parkinson’s disease; HC, healthy control; LEDD, levodopa equivalent daily dosage; H–Y staging, Hoehn–Yahr staging; MDS-UPDRS III, Movement Disorder Society-Unified Parkinson’s Disease Rating Scale part III; BMI, body mass index; SII, systemic immune-inflammation index; SIRI, systemic inflammation response index; NLR, neutrophil-to-lymphocyte ratio; PLR, platelet-to-lymphocyte ratio; MLR, monocyte-to-lymphocyte ratio; NPR, neutrophil-to-platelet ratio; NHR, neutrophil-to-high-density lipoprotein cholesterol ratio; MHR, monocyte-to-high-density lipoprotein cholesterol ratio; LHR, lymphocyte-to-high-density lipoprotein cholesterol ratio; PHR, platelet-to-high-density lipoprotein cholesterol ratio; N/A, not applicable.

**Table 2:** Comparison of laboratory indicators among H–Y staging in PD.

	H–Y < 3 (*n* = 102)	H–Y = 3 (*n* = 132)	H–Y > 3 (*n* = 81)	*p*
SII	384.46 (273.70, 521.25)	374.70 (286.74, 530.58)	443.86 (305.11, 707.70)	**0.021**
SIRI	0.65 (0.50, 0.95)	0.66 (0.46, 1.02)	0.74 (0.51, 1.11)	0.567
NLR	1.92 (1.50, 2.42)	1.93 (1.53, 2.55)	2.22 (1.56, 2.73)	0.103
PLR	117.13 (97.05, 152.15)	124.14 (95.92, 148.62)	138.22 (105.99, 175.93)	**0.011**
MLR	0.22 (0.17, 0.28)	0.21 (0.16, 0.28)	0.23 (0.18, 0.30)	0.383
NPR	0.02 (0.01, 0.02)	0.02 (0.01, 0.02)	0.02 (0.01, 0.02)	0.655
NHR	2.74 (2.04, 3.75)	2.70 (2.10, 3.62)	2.70 (1.97, 3.86)	0.980
MHR	0.31 (0.23, 0.42)	0.31 (0.22, 0.42)	0.30 (0.21, 0.40)	0.719
LHR	1.48 (1.05, 2.08)	1.37 (1.13, 1.73)	1.38 (1.00, 1.72)	0.167
PHR	173.48 (148.41, 214.02)	167.12 (137.95, 204.70)	174.36 (142.11, 218.57)	0.527

Abbreviations: PD, Parkinson’s disease; H–Y, Hoehn–Yahr; SII, systemic immune-inflammation index; SIRI, systemic inflammation response index; NLR, neutrophil-to-lymphocyte ratio; PLR, platelet-to-lymphocyte ratio; MLR, monocyte-to-lymphocyte ratio; NPR, neutrophil-to-platelet ratio; NHR, neutrophil-to-high-density lipoprotein cholesterol ratio; MHR, monocyte-to-high-density lipoprotein cholesterol ratio; LHR, lymphocyte-to-high-density lipoprotein cholesterol ratio; PHR, platelet-to-high-density lipoprotein cholesterol ratio. Statistically significant results are shown in bold.

**Table 3:** Multinomial logistic regression analysis of factors associated with H–Y staging in PD (primary analysis).

	H–Y < 3 vs. H–Y > 3 OR (95% CI)	*p*	H–Y = 3 vs. H–Y > 3 OR (95% CI)	*p*
Age (per year)	0.974 (0.939, 1.010)	0.158	0.985 (0.950, 1.020)	0.391
Sex (male vs. female)	1142 (0.539, 2.422)	0.729	0.832 (0.413, 1.675)	0.607
BMI (per unit)	1.027 (0.951, 1.109)	0.504	0.917 (0.846, 0.994)	**0.035**
Disease duration (per year)	0.940 (0.894, 0.989)	**0.016**	0.961 (0.925, 0.999)	**0.043**
LEDD (per mg/day)	0.999 (0.998, 1.000)	0.050	1.000 (0.999, 1.001)	0.563
Hypertension (yes vs. no)	0.468 (0.226, 0.970)	**0.041**	0.729 (0.373, 1.424)	0.354
Diabetes (yes vs. no)	1.192 (0.448, 3.173)	0.725	0.775 (0.301, 1.995)	0.597
Smoking (yes vs. no)	1.024 (0.446, 2.355)	0.955	0.929 (0.414, 2.084)	0.859
Drinking (yes vs. no)	1.313 (0.541, 3.186)	0.616	0.765 (0.317, 1.850)	0.553
Antiplatelet agents (yes vs. no)	0.649 (0.009, 1.585)	0.051	1.311 (0.842, 2.259)	**0.012**
SII (per unit)	1.000 (0.998, 1.001)	0.723	1.000 (0.998, 1.001)	0.905
PLR (per unit)	0.993 (0.984, 1.003)	0.167	0.989 (0.980, 0.997)	**0.012**

Reference category: H–Y > 3. Model adjusted for age, sex, BMI, LEDD, hypertension, diabetes, smoking, drinking, and antiplatelet agents. Statistically significant results are shown in bold. Abbreviations: PD, Parkinson’s disease; H–Y, Hoehn–Yahr; SII, systemic immune-inflammation index; PLR, platelet-to-lymphocyte ratio; LEDD, levodopa equivalent daily dosage; BMI, body mass index; OR, odds ratio; CI, confidence interval.

**Table 4:** Multivariable linear regression analysis of associations between inflammatory indices and clinical symptoms in PD patients (fully adjusted models).

Inflammatory Index	MDS-UPDRS III B (95% CI)	MoCA ^#^ B (95% CI)	HAMA B (95% CI)	HAMD B (95% CI)
SII	0.007 (−0.000, 0.015)	**−0.003** (**−0.005, 0.000**) *	0.004 (0.000, 0.007)	**0.006** (**0.001, 0.010**) *
SIRI	−0.108 (−1.441, 1.194)	**−0.948** (**−1.881, −0.014**) *	0.657 (−0.959, 2.272)	0.606 (−1.288, 2.500)
PLR	**0.049** (**0.008, 0.091**) *	−0.013 (−0.026, 0.001)	**0.024** (**0.003, 0.045**) *	**0.034** (**0.009, 0.059**) **
MLR	−4.938 (−23.491, 13.616)	−5.448 (−11.199, 0.302)	5.911 (−4.907, 16.729)	4.541 (−8.202, 17.284)
PHR	0.022 (−0.008, 0.052)	−0.009 (−0.019, 0.000)	0.006 (−0.009, 0.021)	0.009 (−0.008, 0.027)

All models were adjusted for age, sex, disease duration, and LEDD. **^#^**, MoCA models were additionally adjusted for years of education. Data are presented as B (95% CI). Statistically significant results are shown in bold. *, *p* < 0.05 (unadjusted for multiple comparisons); **, *p* < 0.01 (unadjusted for multiple comparisons). After Benjamini–Hochberg FDR correction across all 40 tests, no associations remained significant (all q > 0.05, see Supplementary Table S5). Abbreviations: PD, Parkinson’s disease; MDS-UPDRS III, Movement Disorder Society-Unified Parkinson’s Disease Rating Scale part III; MoCA, Montreal Cognitive Assessment; HAMA, Hamilton Anxiety Scale; HAMD, Hamilton Depression Scale; SII, systemic immune-inflammation index; SIRI, systemic inflammation response index; PLR, platelet-to-lymphocyte ratio; MLR, monocyte-to-lymphocyte ratio; PHR, platelet-to-high-density lipoprotein cholesterol ratio; LEDD, levodopa equivalent daily dosage; B, unstandardized regression coefficient; CI, confidence interval.

## Data Availability

The raw clinical and laboratory data used in this study are not publicly available due to patient privacy and ethical restrictions. De-identified datasets that support the findings of this study are available from the corresponding author upon reasonable request.

## Supplementary Materials

The following supporting information can be downloaded at: https://www.mdpi.com/article/10.3390/brainsci16101133/s1, Table S1: FDR-adjusted q-values for comparisons of inflammatory indices; Table S2: Formal tests for PD/HC group × sex interaction for inflammatory indices with significant differences in Figure 2; Table S3: Multinomial logistic regression analysis of the other eight inflammatory indicators with H-Y staging in PD; Table S4: Univariate and minimally adjusted (age + sex) linear regression analyses of inflammatory indices with clinical symptoms in PD; Table S5: FDR-adjusted q-values for associations between inflammatory indices and clinical symptoms (corresponding to Table 4).

## Abbreviations

The following abbreviations are used in this manuscript:

PD	Parkinson’s disease
HC	Healthy control
BMI	Body mass index
H–Y staging	Hoehn–Yahr staging
MDS-UPDRS III	Movement Disorder Society-Unified Parkinson’s Disease Rating Scale part III
MoCA	Montreal Cognitive Assessment
HAMA	Hamilton Anxiety Scale
HAMD	Hamilton Depression Scale
LEDD	Levodopa equivalent daily dosage
SII	Systemic immune-inflammation index
SIRI	Systemic inflammation response index
NLR	Neutrophil-to-lymphocyte ratio
PLR	Platelet-to-lymphocyte ratio
MLR	Monocyte-to-lymphocyte ratio
NPR	Neutrophil-to- platelet ratio
NHR	Neutrophil-to-high-density lipoprotein cholesterol ratio
MHR	Monocyte-to-high-density lipoprotein cholesterol ratio
LHR	Lymphocyte-to-high-density lipoprotein cholesterol ratio
PHR	Platelet-to-high-density lipoprotein cholesterol ratio

## References

[1] Su D., Cui Y., He C., Yin P., Bai R., Zhu J., Lam J.S.T., Zhang J., Yan R., Zheng X. (2025). Projections for prevalence of Parkinson’s disease and its driving factors in 195 countries and territories to 2050: Modelling study of Global Burden of Disease Study 2021. BMJ.

[2] Araújo B., Caridade-Silva R., Soares-Guedes C., Martins-Macedo J., Gomes E.D., Monteiro S., Teixeira F.G. (2022). Neuroinflammation and Parkinson’s Disease-From Neurodegeneration to Therapeutic Opportunities. Cells.

[3] Isik S., Yeman Kiyak B., Akbayir R., Seyhali R., Arpaci T. (2023). Microglia Mediated Neuroinflammation in Parkinson’s Disease. Cells.

[4] Deyell J.S., Sriparna M., Ying M., Mao X. (2023). The Interplay between α-Synuclein and Microglia in α-Synucleinopathies. Int. J. Mol. Sci..

[5] Kamil S., Natalia S., Grzegorz G., Aleksandra K.-L., Wioletta R.-K., Ireneusz M. (2024). Alpha-Synuclein Contribution to Neuronal and Glial Damage in Parkinson’s Disease. Int. J. Mol. Sci..

[6] Tizibt Ashine B., Domenico M., Alessia V., Serena S., Gaia F., Francesca L., Aurora B., Stefania M., Arianna B., Stefano F. (2025). Experimental Ischemic Stroke-Induced Alpha-Synuclein Pathology Enhances Endothelial Inflammatory Response and Impairs Angiogenesis. Stroke.

[7] Nepozitek J., Marecek S., Rottova V., Dostalova S., Krajca T., Keller J., Sonka K., Dusek P. (2025). Glymphatic dysfunction evidenced by DTI-ALPS is related to obstructive sleep apnea intensity in newly diagnosed Parkinson’s disease. npj Park. Dis..

[8] Yue Y., Zhang X., Lv W., Lai H.Y., Shen T. (2024). Interplay between the glymphatic system and neurotoxic proteins in Parkinson’s disease and related disorders: Current knowledge and future directions. Neural Regen. Res..

[9] Ding X.B., Wang X.X., Xia D.H., Liu H., Tian H.Y., Fu Y., Chen Y.K., Qin C., Wang J.Q., Xiang Z. (2021). Impaired meningeal lymphatic drainage in patients with idiopathic Parkinson’s disease. Nat. Med..

[10] Islam M.M., Satici M.O., Eroglu S.E. (2024). Unraveling the clinical significance and prognostic value of the neutrophil-to-lymphocyte ratio, platelet-to-lymphocyte ratio, systemic immune-inflammation index, systemic inflammation response index, and delta neutrophil index: An extensive literature review. Turk. J. Emerg. Med..

[11] Nazir S., Jankowski V., Bender G., Zewinger S., Rye K.A., van der Vorst E.P.C. (2020). Interaction between high-density lipoproteins and inflammation: Function matters more than concentration!. Adv. Drug Deliv. Rev..

[12] Liu Z., Fan Q., Wu S., Wan Y., Lei Y. (2021). Compared with the monocyte to high-density lipoprotein ratio (MHR) and the neutrophil to lymphocyte ratio (NLR), the neutrophil to high-density lipoprotein ratio (NHR) is more valuable for assessing the inflammatory process in Parkinson’s disease. Lipids Health Dis..

[13] Kwak I.H., Kim Y.E., Kim Y.J., Noh H.M., Lee J., Yu J.K., Ma H.I. (2024). Monocyte to high-density lipoprotein cholesterol ratio reflects the peripheral inflammatory state in parkinsonian disorders. Park. Relat. Disord..

[14] Zhao J., Wu Z., Cai F., Yu X., Song Z. (2025). Higher systemic immune-inflammation index is associated with increased risk of Parkinson’s disease in adults: A nationwide population-based study. Front. Aging Neurosci..

[15] Liu F., Ran Q., Zhang H., Chen J. (2025). The Systemic Immune-Inflammation Index and the Risk of Parkinson’s Disease in the U.S.: A Cross-Sectional Study. J. Clin. Med..

[16] Deussen A., Kopaliani I. (2023). Targeting inflammation in hypertension. Curr. Opin. Nephrol. Hypertens..

[17] Rao P.P., Mishra S., Gupta J., Vyas M., Babu M.R. (2025). Inflammation and immune biomarkers: New frontiers in understanding and managing diabetes complications. Inflammopharmacology.

[18] Stanca I.D., Criciotoiu O., Neamtu S.D., Vasile R.C., Berceanu-Bora N.M., Minca T.N., Pirici I., Rosu G.C., Bondari S. (2022). The Analysis of Blood Inflammation Markers as Prognostic Factors in Parkinson’s Disease. Healthcare.

[19] Li F., Wang Z., Deng M., Peng J., He G., Tong Y., Xu W., Feng T., Song K. (2026). Correlation of systemic immune inflammation index and systemic inflammatory response index with the severity of Parkinson’s disease. Front. Neurol..

[20] Benchimol E.I., Smeeth L., Guttmann A., Harron K., Moher D., Petersen I., Sørensen H.T., von Elm E., Langan S.M. (2015). The REporting of studies Conducted using Observational Routinely-collected health Data (RECORD) statement. PLoS Med..

[21] Postuma R.B., Berg D., Stern M., Poewe W., Olanow C.W., Oertel W., Obeso J., Marek K., Litvan I., Lang A.E. (2015). MDS clinical diagnostic criteria for Parkinson’s disease. Mov. Disord..

[22] Tomlinson C.L., Stowe R., Patel S., Rick C., Gray R., Clarke C.E. (2010). Systematic review of levodopa dose equivalency reporting in Parkinson’s disease. Mov. Disord..

[23] Jiang L., Zhong Z., Huang J., Bian H., Huang W. (2022). Monocytohigh-density lipoprotein ratio has a high predictive value for the diagnosis of multiple system atrophy and the differentiation from Parkinson’s disease. Front. Aging Neurosci..

[24] Kwak I.H., Ma H.I., Kim Y.J., Noh H.M., Lee J., Yu J.K., Kim Y.E. (2025). Peripheral inflammatory markers and clinical phenotypes reflecting the impact of diabetes on Parkinson’s disease. J. Park. Dis..

[25] Alagoz A.N., Dagdas A., Bunul S.D., Erci G.C. (2025). The Relationship Between Systemic Inflammatory Index and Other Inflammatory Markers with Clinical Severity of the Disease in Patients with Parkinson’s Disease. Biomedicines.

[26] Pan H., Wang X., Zhang X., Shi X., Sun T., Xu J., Wang S. (2025). Investigating the association of pan-immune-inflammation value, systemic immune-inflammation index, and neutrophil-to-lymphocyte ratio with pain in Parkinson’s disease. Front. Neurol..

[27] Boots E.A., Castellanos K.J., Zhan L., Barnes L.L., Tussing-Humphreys L., Deoni S.C.L., Lamar M. (2020). Inflammation, Cognition, and White Matter in Older Adults: An Examination by Race. Front. Aging Neurosci..

[28] Bovenzi R., Conti M., Simonetta C., Bissacco J., Mascioli D., Mancini M., Buttarazzi V., Veltri F., Sancesario G.M., Bagetta S. (2025). Sex-specific immune-biological profiles in Parkinson’s disease. J. Neuroimmunol..

[29] Jiang G.Y., Li F., Yin J.H., Cao L.X. (2026). Peripheral Inflammatory Biomarkers in Parkinson’s Disease: Clinical Correlations and Stratification. Cell. Mol. Neurobiol..

[30] Li H.J., Yu Y., Chen Y., Liang H.Y. (2015). Vascular risk factors aggravate the progression of Parkinson’s disease: A five-year follow-up study in Chinese patients. Int. J. Clin. Exp. Med..

[31] Johri M., Morrill V., Okine D.N., Huang X., Rosenthal L., Seemiller J., Shrestha S., Sullivan K.J., Chen H., Gottesman R.F. (2025). Midlife Vascular Risk Factors, Parkinson Disease, and Parkinson Disease-Dementia: The ARIC Study. Neurology.

[32] Sheikh A.M., Yano S., Tabassum S., Nagai A. (2024). The Role of the Vascular System in Degenerative Diseases: Mechanisms and Implications. Int. J. Mol. Sci..

[33] Atasever E., Cebe T., Turgut Ş., Andican G., Çakatay U. (2026). Cytoskeletal disintegration in cardiovascular disease-related neurodegeneration. Adv. Protein Chem. Struct. Biol..

[34] Kaya M.G., Yarlioglues M., Gunebakmaz O., Gunturk E., Inanc T., Dogan A., Kalay N., Topsakal R. (2010). Platelet activation and inflammatory response in patients with non-dipper hypertension. Atherosclerosis.

[35] Du X.J., Kiriazis H. (2013). Pro-inflammatory role of platelets in hypertension-mediated end-organ damage: Editorial to: “Inhibition of Platelet activation by clopidogrel prevents hypertension-induced cardiac inflammation and fibrosis” by L.-X. Jia et al. Cardiovasc. Drugs Ther..

[36] Zhou W., Zeng T., Liu D., Pang R., Gong L. (2025). Association between inflammatory markers (SII and SIRI) and anxiety levels in Parkinson’s disease. Front. Psychiatry.

[37] Yang D., Liu Z., Lin S., Wang J., Zhang S., Xu J., Cai H., Zhang B., Lin H., Huang S. (2026). Neutrophil-to-Lymphocyte Ratio Predicts Anxiety Progression in Parkinson’s Disease. Mov. Disord. Clin. Pract..

[38] He P., Li Y., Huang Z., Gao Y., Duan Q., Qiu Y., Feng S., Huang R., Gong L., Ma G. (2025). Peripheral inflammation’s variable impact on cognitive and symptomatic outcomes in Parkinson’s disease: A longitudinal and cross-sectional analysis. npj Park. Dis..

[39] Lucero J., Gurnani A., Weinberg J., Shih L.C. (2024). Neutrophil-to-lymphocyte ratio and longitudinal cognitive performance in Parkinson’s disease. Ann. Clin. Transl. Neurol..

[40] Gibbons C., Wang N., Rajan S., Kern D., Palma J.A., Kaufmann H., Freeman R. (2023). Cutaneous α-Synuclein Signatures in Patients with Multiple System Atrophy and Parkinson Disease. Neurology.

[41] Wang Z., Becker K., Donadio V., Siedlak S., Yuan J., Rezaee M., Incensi A., Kuzkina A., Orrú C.D., Tatsuoka C. (2020). Skin α-Synuclein Aggregation Seeding Activity as a Novel Biomarker for Parkinson Disease. JAMA Neurol..

[42] Donadio V., Ingelsson M., Rizzo G., Furia A., Incensi A., Delprete C., Pinho M., Liguori R., Pritzkow S. (2025). Diagnostic biomarkers for α-synucleinopathies-state of the art and future developments: A systematic review. Mol. Neurodegener..

[43] Zhao Z., Fu Q., Guo X., He H., Yang G. (2025). Potential Biomarkers and Treatment of Neuroinflammation in Parkinson’s Disease. Actas Esp. Psiquiatr..

