# Supplementary material for: Systemic Inflammatory Markers in Parkinson’s Disease: Correlations with Disease Severity and Neuropsychiatric Symptoms
Source: Brain Sci. 2026 Oct 20;16(10):1133. doi: 10.3390/brainsci16101133 (PMC13702398; doi:10.3390/brainsci16101133)
Supplement: Supplementary file 1 [file brainsci-16-01133-s001.zip › brainsci-4594741-supplementary.pdf]

Supplementary Table S1 FDR-adjusted q-values for comparisons of inflammatory indices

	p (PD vs HC)	q (FDR-adjusted)	p (H-Y groups)	q (FDR-adjusted)
SII	0.090	0.162	**0.021**	0.105
SIRI	**0.005**	**0.017**	0.567	0.799
NLR	0.304	0.380	0.103	0.343
PLR	0.627	0.627	**0.011**	0.105
MLR	**< 0.001**	**< 0.001**	0.383	0.766
NPR	0.087	0.162	0.655	0.799
NHR	0.097	0.162	0.980	0.980
MHR	**< 0.001**	**< 0.001**	0.719	0.799
LHR	0.176	0.251	0.167	0.418
PHR	0.348	0.387	0.527	0.799

FDR correction was applied using the Benjamini-Hochberg method across the ten inflammatory indices. $q < 0.05$ was considered significant.

Abbreviations: PD, Parkinson's disease; HC, healthy controls; SII, systemic immune-inflammation index; SIRI, systemic inflammation response index; NLR, neutrophil-to-lymphocyte ratio; PLR, platelet-to-lymphocyte ratio; MLR, monocyte-to-lymphocyte ratio; NPR, neutrophil-to- platelet ratio; NHR, neutrophil-to-high-density lipoprotein cholesterol ratio; MHR, monocyte-to-high-density lipoprotein cholesterol ratio; LHR, lymphocyte-to-high-density lipoprotein cholesterol ratio; PHR, platelet-to-high-density lipoprotein cholesterol ratio

Supplementary Table S2 Formal tests for PD/HC group × sex interaction for inflammatory indices with significant differences in Figure 2

	Group × sex interaction B (SE)	p
SIRI	-0.244 (0.317)	0.443
PLR	-15.105 (10.055)	0.134
MLR	-0.042 (0.021)	0.045
NPR	-0.001 (0.001)	0.365
NHR	0.009 (0.270)	0.973
MHR	-0.109 (0.064)	0.090

Models: dependent variable = inflammatory index; predictors = group (0=HC, 1=PD), sex (1=male, 2=female), and group × sex interaction. Given that six interaction tests were performed, we applied Bonferroni correction (threshold $p < 0.008$).

Abbreviations: PD, Parkinson's disease; HC, healthy controls; SIRI, systemic inflammation response index; PLR, platelet-to-lymphocyte ratio; MLR, monocyte-to-lymphocyte ratio; NPR, neutrophil-to- platelet ratio; NHR, neutrophil-to-high-density lipoprotein cholesterol ratio; MHR, monocyte-to-high-density lipoprotein cholesterol ratio; B, unstandardized coefficient; SE, standard error.

Supplementary Table S3 Multinomial logistic regression analysis of the other eight inflammatory indicators with H-Y staging in PD

	H-Y < 3 vs H-Y > 3 OR (95% CI)	p	H-Y < 3 vs H-Y > 3 OR (95% CI)	p
SIRI	0.730 (0.449, 1.042)	0.202	1.016 (0.866, 1.192	0.845
NLR	0.736 (0.541, 1.000)	0.050	0.753 (0.576, 0.985)	**0.039**
MLR	0.165 (0.009, 3.092)	0.228	0.207 (0.019, 2.271)	0.197
NPR (per 0.01-unit increase)*	0.916 (0.567, 1.478)	0.718	1.093 (0.712, 1.678)	0.685
NHR	0.881 (0.696, 1.114)	0.290	0.876 (0.712, 1.078)	0.211
LHR	1.486 (0.858, 2.573)	0.157	1.416 (0.846, 2.369)	0.186
MHR	0.819 (0.124, 5.418)	0.836	0.743 (0.138, 4.005)	0.730
PHR	0.998 (0.994, 1.002)	0.998	0.996 (0.992, 1.000)	0.079

Model adjusted for age, sex, BMI, smoking, drinking, diabetes, and antiplatelet agents. *, NPR was multiplied by 100 for model stability; the OR corresponds to a 0.01-unit increase in NPR.

Abbreviations: PD, Parkinson's disease; H-Y, Hoehn-Yahr; SIRI, systemic inflammation response index; NLR, neutrophil-to-lymphocyte ratio; MLR, monocyte-to-lymphocyte ratio; NPR, neutrophil-to- platelet ratio; NHR, neutrophil-to-high-density lipoprotein cholesterol ratio; MHR, monocyte-to-high-density lipoprotein cholesterol ratio; LHR, lymphocyte-to-high-density lipoprotein cholesterol ratio; PHR, platelet-to-high-density lipoprotein cholesterol ratio; OR, odds ratio; CI, confidence interval.

Supplementary Table S4 Univariate and minimally adjusted (age + sex) linear regression analyses of inflammatory indices with clinical symptoms in PD

A. MDS-UPDRS III

	Unadjusted B (95% CI)	p	Adjusted B (95% CI)	p
SII	0.008 (0.000, 0.015)	0.056	0.008 (0.000, 0.015)	0.055
SIRI	0.016 (-1.314, 1.346)	0.981	0.050 (-1.286, 1.386)	0.074
NLR	0.771 (-1.154, 2.695)	0.788	0.937 (-1.027, 2.900)	0.349
PLR	0.049 (0.007, 0.091)	**0.022**	0.048 (0.005, 0.090)	**0.027**
MLR	-7.066 (-25.150,11.018)	0.443	-5.182 (-24.068, 13.705)	0.590
NPR*	-2.232 (-5.173, 0.709)	0.136	-2.020 (-5.106, 1.067)	0.199
NHR	-0.113 (-1.613, 1.387)	0.882	0.011 (-1.518, 1.541)	0.988
MHR	-8.349 (-20.617, 3.919)	0.182	-7.389 (-20.278, 5.499)	0.260
LHR	-1.524 (-4.960, 1.912)	0.383	-1.454 (-4.911, 2.003)	0.409
PHR	0.020 (-0.011, 0.050)	0.211	0.019 (-0.012, 0.050)	0.232

B. MoCA

	Unadjusted B (95% CI)	p	Adjusted B (95% CI)	p
SII	-0.003 (-0.006, -0.001)	**0.009**	-0.003 (-0.005, 0.000)	**0.032**
SIRI	-1.143 (-2.087, -0.199)	**0.018**	-1.131 (-2.062, -0.200)	**0.017**
NLR	-0.572 (-1.189, 0.045)	0.069	-0.392 (-0.999, 0.214)	0.204
PLR	-0.013 (-0.026, 0.001)	0.070	0.009 (-0.022, 0.004)	0.193

MLR	-5.537 (-11.368, 0.294)	0.063	-6.309 (-12.131, -0.487)	**0.034**
NPR*	0.093 (-0.863, 1.049)	0.849	0.203 (-0.754, 1.160)	0.677
NHR	-0.490 (-0.972, -0.007)	**0.047**	-0.455 (-0.927, 0.017)	0.059
MHR	-2.627 (-6.599, 1.345)	0.194	-3.695 (-7.682, 0.292)	0.069
LHR	0.004 (-1.133, 1.141)	**0.006**	0.240 (-1.340, 0.859)	0.668
PHR	-0.010 (-0.020, 0.000)	**0.046**	-0.011 (-0.020, -0.011)	**0.029**

C. HAMA

	Unadjusted B (95% CI)	p	Adjusted B (95% CI)	p
SII	0.004 (0.000, 0.008)	**0.031**	0.004 (0.000, 0.008)	0.062
SIRI	0,191 (-1.416, 1.798)	0.815	0.702 (-0.911, 2.315)	0.392
NLR	0.633 (-0.361, 1.627)	0.211	0.699 (-0.292, 1.689)	0.166
PLR	0.030 (0.009, 0.051)	**0.006**	0.024 (0.003, 0.045)	**0.028**
MLR	0.555 (-10.006, 11.116)	0.918	5.983 (-4.804, 16.770)	0.276
NPR*	-1.031 (-2.497, 0.435)	0.167	-0.558 (-2.045, 0.930)	0.461
NHR	-0.055 (-0.800, 0.690)	0.885	0.114 (-0.626, 0.854)	0.762
MHR	-3.638 (-9.673, 2.398)	0.236	-0.454 (-6.688, 5.781)	0.887
LHR	-1.062 (-2.829, 0.705)	0.237	-0.645 (-2.391, 1.100)	0.467
PHR	0.007 (-0.009, 0.022)	0.402	0.006 (-0.009, 0.021)	0.457

D. HAMD

	Unadjusted B (95% CI)	p	Adjusted B (95% CI)	p
SII	0.006 (0.002, 0.011)	**0.007**	0.006 (0.001, 0.010)	**0.014**
SIRI	0.012 (-1.874, 1.899)	0.990	0.672 (-1.215, 2.559)	0.484
NLR	0.896 (-0.268, 2.061)	0.131	1.011 (-0.144, 2.166)	0.086
PLR	0.042 (0.017, 0.066)	**0.001**	0.034 (0.010, 0.059)	**0.006**
MLR	-1.825 (-14.258, 10.609)	0.773	4.992 (-7.742, 17.587)	0.445
NPR*	-1.193 (-2.913, 0.527)	0.173	-0.561 (-2.301, 1.180)	0.526
NHR	0.038 (-0.836, 0.912)	0.932	0.264 (-0.603, 1.130)	0.549
MHR	-5.748 (-12.807, 1.312)	0.110	-1.868 (-9.148, 5.411)	0.614
LHR	-1.540 (-3.634, 0.554)	0.149	-1.036 (-3.098, 1.025)	0.323
PHR	0.010 (-0.008, 0.028)	0.272	0.009 (-0.009, 0.027)	0.323

*, NPR was multiplied by 100 for model stability. Statistically significant results are shown in bold.

Abbreviations: PD, Parkinson's disease; H-Y, Hoehn-Yahr; MDS-UPDRS III, Movement Disorder Society-Unified Parkinson's Disease Rating Scale part III; MoCA, Montreal Cognitive Assessment; HAMA, Hamilton Anxiety Scale; HAMD, Hamilton Depression Scale; SII, systemic immune-inflammation index; SIRI, systemic inflammation response index; NLR, neutrophil-to-lymphocyte ratio; PLR, platelet-to-lymphocyte ratio; MLR, monocyte-to-lymphocyte ratio; NPR, neutrophil-to-platelet ratio; NHR, neutrophil-to-high-density lipoprotein cholesterol ratio; MHR, monocyte-to-high-density lipoprotein cholesterol ratio; LHR, lymphocyte-to-high-density lipoprotein cholesterol ratio; PHR, platelet-to-high-density lipoprotein cholesterol ratio; B, unstandardized coefficient.

Supplementary Table S5 FDR-adjusted q-values for associations between inflammatory indices and clinical symptoms (corresponding to Table 4)

	MDS-UPDRS III		MoCA		HAMA		HAMD	
	p	q	p	q	p	q	p	q
SII	0.067	0.262	0.039	0.262	0.072	0.262	0.017	0.262
SIRI	0.870	0.912	0.047	0.262	0.424	0.684	0.529	0.684
NLR	0.425	0.684	0.219	0.515	0.183	0.458	0.097	0.323
PLR	0.020	0.262	0.062	0.262	0.028	0.262	0.007	0.262
MLR	0.601	0.695	0.063	0.262	0.283	0.571	0.483	0.684
NPR*	0.146	0.446	0.608	0.695	0.452	0.684	0.530	0.684
NHR	0.997	0.997	0.169	0.451	0.773	0.836	0.562	0.695
MHR	0.298	0.571	0.251	0.558	0.889	0.912	0.591	0.695
LHR	0.490	0.684	0.763	0.836	0.510	0.684	0.360	0.655
PHR	0.156	0.446	0.063	0.262	0.441	0.684	0.300	0.571

Models adjusted for age, sex, disease duration, and LEDD; MoCA models additionally adjusted for years of education. FDR correction was applied across all 40 tests.

*, NPR was multiplied by 100 for model stability

Abbreviations: MDS-UPDRS III, Movement Disorder Society-Unified Parkinson's Disease Rating Scale part III; MoCA, Montreal Cognitive Assessment; HAMA, Hamilton Anxiety Scale; HAMD, Hamilton Depression Scale; SII, systemic immune-inflammation index; SIRI, systemic inflammation response index; PLR, platelet-to-lymphocyte ratio; MLR, monocyte-to-lymphocyte ratio; PHR, platelet-to-high-density lipoprotein cholesterol ratio.
